# Cholesterol metabolism and serum non-cholesterol sterols: summary of 13 plant stanol ester interventions

**DOI:** 10.1186/1476-511X-13-72

**Published:** 2014-04-27

**Authors:** Maarit Hallikainen, Piia Simonen, Helena Gylling

**Affiliations:** 1 Institute of Public Health and Clinical Nutrition, Department of Clinical Nutrition, University of Eastern Finland, Kuopio, Finland; 2 Heart and Lung Center, Helsinki University Hospital, Helsinki, Finland; 3 Department of Medicine, Division of Internal Medicine, University of Helsinki, Helsinki, Finland, P.O. BOX 700 FIN-00029 HUS, Finland

**Keywords:** LDL cholesterol, Cholesterol absorption, Cholesterol synthesis, Cholestanol, Campesterol, Sitosterol, Lathosterol, Desmosterol, Squalene

## Abstract

**Background:**

The efficacy and safety of plant stanols added to food products as serum cholesterol lowering agents have been demonstrated convincingly, but their effects on cholesterol metabolism and on serum non-cholesterol sterols is less evaluated. The aim of this study was to assess the validity of serum non-cholesterol sterols and squalene as bioindices of cholesterol synthesis and absorption, and to examine how the individual serum non-cholesterol sterols respond to consumption of plant stanols.

**Methods:**

We collected all randomized, controlled plant stanol ester (STAEST) interventions in which serum cholestanol, plant sterols campesterol and sitosterol, and at least two serum cholesterol precursors had been analysed. According to these criteria, there was a total of 13 studies (total 868 subjects without lipid-lowering medication; plant stanol doses varied from 0.8 to 8.8 g/d added in esterified form; the duration of the studies varied from 4 to 52 weeks). Serum non-cholesterol sterols were assayed with gas–liquid chromatography, cholesterol synthesis with the sterol balance technique, and fractional cholesterol absorption with the dual continuous isotope feeding method.

**Results:**

The results demonstrated that during the control and the STAEST periods, the serum plant sterol/cholesterol- and the cholestanol/cholesterol-ratios reflected fractional cholesterol absorption, and the precursor sterol/cholesterol-ratios reflected cholesterol synthesis. Plant sterol levels were dose-dependently reduced by STAEST so that 2 g of plant stanols reduced serum campesterol/cholesterol-ratio on average by 32%. Serum cholestanol/cholesterol-ratio was reduced less frequently than those of the plant sterols by STAEST, and the cholesterol precursor sterol ratios did not change consistently in the individual studies emphasizing the importance of monitoring more than one surrogate serum marker.

**Conclusions:**

Serum non-cholesterol sterols are valid markers of cholesterol absorption and synthesis even during cholesterol absorption inhibition with STAEST. Serum plant sterol concentrations decrease dose-dependently in response to plant stanols suggesting that the higher the plant stanol dose, the more cholesterol absorption is inhibited and the greater the reduction in LDL cholesterol level is that can be achieved.

**Trial registration:**

Clinical Trials Register # NCT00698256 [Eur J Nutr 2010, 49:111-117]

## Background

The hypocholesterolemic effect of dietary plant sterol supplementation was demonstrated for the first time in humans in the 1950′s [1] and that of plant stanols in the 1980′s [2]. Today, several food products with added plant sterols and plant stanols are used worldwide as a dietary means to lower serum total and LDL cholesterol concentrations by interfering with cholesterol absorption [3,4]. Thus, in addition to the reduction in the serum cholesterol concentration, these agents makes it possible to evaluate cholesterol metabolism in response to inhibition of cholesterol absorption.

The assessment of cholesterol absorption and synthesis is laborious in clinical studies. For this reason, serum non-cholesterol sterols have been examined as relative markers of whole-body cholesterol metabolism under steady state conditions with subjects consuming a normal habitual diet. The levels of serum cholesterol precursors (squalene, cholestenol, desmosterol, and lathosterol) reflect the activity of cholesterol synthesis, and serum plant sterols (campesterol and sitosterol), and cholestanol, reflect the absorption efficiency of cholesterol. Their validity has been assessed by comparing the synthesis marker values with those obtained with the sterol balance technique [5-11] or with the evaluation of hepatic 3-hydroxy-3-methyl-glutaryl-CoA reductase activity [12], and the absorption marker values have been validated with those obtained with continuous peroral or peroral-intravenous isotopes and tracers [6-9,13,14]. For particular, the cholesterol-standardized ratios of the relevant serum markers were best associated with cholesterol synthesis and absorption efficiency [6-9], and the absorption marker/synthesis marker ratios with whole-body cholesterol metabolism [6,7]. However, especially during interventions and when only one serum marker has been used, there have been inconsistencies in the results between the serum markers and absolute methods [8,11]. In a recent cross-sectional study, e.g., serum plant sterol/cholesterol-ratios did not correlate with fractional cholesterol absorption [15]. In this study, the study population of 175 hypercholesterolemic subjects was divided into those with the lowest and highest campesterol/cholesterol ratio, and cholesterol absorption efficiency was measured with a single oral-intravenous dose. Cholesterol absorption efficiency did not differ between the groups. Of special interest was the very low values of cholesterol absorption efficiency with a mean of 24%, and only a few subjects reached the value of 50%, which is considered the average mean value among different populations. The results were discussed by Grundy [16] that there may be methodological reasons related to the tracer or the single dose-method per se causing these particularly low cholesterol absorption values, which could explain the lack of association. To this end, the aim of this study was to assess the validity of the cholesterol absorption and synthesis markers during consumption of plant stanol-supplemented food products. We also examined the changes in cholesterol metabolism occurring in response to plant stanol interventions. Since in general the number of subjects in single plant stanol interventions has been somewhat limited, we gathered together from PubMed all randomized controlled studies in adults, in which at least two serum synthesis markers, two serum plant sterols, and serum cholestanol levels were analysed during plant stanol intervention.

## Methods

### Study population

With these criteria, 13 studies could be identified. These included 868 subjects (414 men and 454 women) with an age range from 20 to 73 years (Table 1). In eleven of the studies, the subjects had mild to moderate hypercholesterolemia [17-27]. In one study, the subjects were type 1 diabetics [28], and another study focused on type 2 diabetics [29]. All subjects were without lipid-lowering medication. The plant stanol dose varied from 0.8 to 8.8 g/d, and in all of the studies, the plant stanols were consumed as their fatty acid esters. Two of the studies were dose–response studies [23,27]. Six studies had a cross-over and seven employed a parallel design. The duration of plant stanol ester (STAEST) consumption varied from 4 to 52 weeks.

**Table 1:** LDL cholesterol and serum non-cholesterol sterol/cholesterol-ratios in different studies during STAEST consumption

					**First value: mean control value. In parenthesis: net relative change between STAEST and control values**							
**Reference**	**Design**	**N**	**Dose (g/d)**	**Duration (wk)**	**LDL-C**	**Cholestanol**	**Campesterol**	**Sitosterol**	**Squalene**	**Cholestenol**	**Desmosterol**	**Lathosterol**
Ref [18]	Parallel	189	2	52	3.9 **(−8%)**	152 (−5%)	301 **(−35%)**	156 **(−37%)**	16 (−1%)	21 **(7%)**	81 **(7%)**	121 **(7%)**
Ref [24]	Crossover	34	2	4	4.2 **(−13%)**	136 (−4%)	388 **(−28%)**	172 **(−29%)**	33 **(22%)**	16 **(23%)**	61 **(11%)**	164 **(20%)**
Ref [22]	Parallel	76	2	10	3.5 **(−9%)**	155 (0%)	278 **(−33%)**	129 **(−35%)**	17 (4%)	17 (10%)	82 **(9%)**	129 **(16%)**
Ref [25]	Parallel	190	2	12	3.9 **(−9%)**	151 **(−4%)**	284 **(−29%)**	142 **(−32%)**	18 (5%)	20 **(19%)**	98 (3%)	137 **(19%)**
Ref [28]	Parallel	19	2	12	3.1**(−16%)**	178 (−4%)	483 **(−24%)**	198 **(−23%)**	17 (−30%)	18 (2%)	73 (0%)	95 (−3%)
Ref [19]	Crossover	21	2.4	5	4.2 **(−12%)**	123 **(−9%)**	209 (−**28%)**	126 **(−28%)**	45 (11%)	18 (**14%)**	74 **(15%)**	175 **(16%)**
Ref [29]	Crossover	11	3	6	3.8 **(−9%)**	94 **(−10%)**	241 **(−43%)**	110 **(−42%)**	31 (13%)	32 **(10%)**	109 **(12%)**	185 **(14%)**
Ref [21]	Parallel	153	3	26	4.1 **(−11%)**	120 **(−8%)**	316 **(−39%)**	152 **(−18%)**	34 (2%)	18 **(34%)**	71 **(11%)**	176 **(20%)**
Ref [20]	Crossover	22	3	7	3.7 **(−17%)**	127 **(−9%)**	301 **(−41%)**	147 **(−22%)**	36 (6%)	15 **(19%)**	91 **(11%)**	170 **(12%)**
Ref [26]	Parallel	67	3.4	6	3.7 **(−9%)**	122 (−3%)^1^	346 **(−48%)**^1^	151 **(−41%)**^1^	33 (13%)^1^	19 **(21%)**	111 **(27%)**	203 **(10%)**
Ref [17]	Parallel	49	8.8	10	3.2 **(−17%)**	157 (−2%)	337 **(−62%)**	164 **(−48%)**	15 (14%)	23 (20%)	86 (15%)	130 (30%)
*Dose–response studies*												
Ref [27]	Crossover	15	0.8	9	4.4 (−8%)	102 (4%)^1^	360 **(−28%)**^1^	163 **(−24%)**^1^	18 (14%)^1^	20 (−5%)^1^	69 (1%)^1^	162 (2%)^1^
			2	6	4.5 **(−15%)**	102 (−2%)^1^	347 **(−48%)**^1^	158 **(−41%)**^1^	20 (−4%)^1^	22 (10%)^1^	28 (2%)^1^	160**(16%)**^1^
Ref [23]	Crossover	22	0.8	4	4.4 (−2%)	141 (1%)	354 **(−20%)**	167 **(−39%)**	39 (9%)	21 (3%)	89 (4%)	133 (13%)
			1.6		“**(−6%)**	”(−1%)	”**(−35%)**	”**(−21%)**	”(14%)	”(18%)	”(6%)	”**(18%)**
			2.3		“**(−10%)**	”(1%)	”**(−35%)**	”**(−34%)**	”(21%)	”**(22%)**	”(7%)	”**(19%)**
			3.1		“**(−10%)**	”(1%)	”**(−39%)**	”**(−32%)**	”(21%)	”(12%)	”(8%)	”**(19%)**

Plant stanols (g/d) added as plant stanol ester (STAEST) to the test mayonnaise [26,27], test spread [18-25,28,29] or test spread and test oat-based drink [17].

LDL is given as mmol/l, and squalene- and non-cholesterol sterol/cholesterol-ratios as 10^2^ × μmol/mmol of cholesterol.

^1^Calculated from the mean values given in the publication.

Bold: p < 0.05 compared with control.

All subjects gave their written informed consent. All studies were performed according to the principles of the Declaration of Helsinki. The Ethics Committees of the University of Helsinki, Second Department of Medicine (studies in ref 19,20,21,26,27,29), University of Kuopio (studies in ref 17,22,23,24,28), the North Karelia Central Hospital (study in ref 18), and Joint Ethics Committee of the University of Turku and Turku University Central Hospital (study in ref 25) had approved the study protocols.

### Measurements

Serum concentrations of non-cholesterol sterols were quantified from non-saponifiable serum-based materials by capillary gas–liquid chromatography (GLC) (Agilent 6890 N Network GC System, Agilent Technologies, Wilmington, DE) equipped with a 50 m long non-polar Ultra 2 capillary column (5% phenyl-methyl siloxane; Agilent Technologies, Wilmington, DE) with 5α-cholestane as internal standard [30]. Typical CVs at relevant concentrations were: cholesterol 3.2%, cholestanol 2.7%, desmosterol 6.0%, lathosterol 3.7%, campesterol 1.8%, and sitosterol 2.4%. Serum non-cholesterol sterols are given as concentrations (μg/dl) and in terms of ratios to cholesterol (10^2^ × μmol/mmol of cholesterol), which was obtained from the same GLC run.

In the absolute metabolic studies, the participants took a capsule containing 200 mg of Cr_2_O_3_, ^3^H-sitostanol, and ^14^C-cholesterol three times a day with each major meal for a week. During the last three days, stools were collected and pooled for analysis of labels, neutral sterols, and bile acids. The sterols and bile acids were measured with GLC [31], using the recovery of Cr_2_O_3_ or labeled sitostanol for measurement of fecal flow. During the week, the participants kept a food diary so that energy, fat and cholesterol intakes could be quantified with the assistance of a computerized program [32].

### Statistics

All statistical analyses were performed with the SPSS for Windows 19.0 statistics program (SPSS, Chicago, IL, USA).

Since the separate studies differed in their design (Table 1), the relative changes from the control values were recalculated from original data except in two studies [26,27], in which the relative changes were calculated from the mean values given in the publications. The normal distribution of variables was confirmed before further statistical analyses. When detecting the relationships between the plant stanol dose and serum LDL cholesterol and non-cholesterol sterols, nine studies with hypercholesterolemic subjects [17-25] were included into the further statistical analyses. Curve equations were calculated by analysis of curve estimation and ANCOVA with the study as a covariate. Pearson correlation coefficients were also calculated. In addition, when detecting the validity, i.e. the associations between the simultaneous measurements of the serum non-cholesterol sterols and absolute cholesterol synthesis and absorption, the Pearson or Spearman correlation coefficients were calculated from the studies in which the data was available [20,27,29]. A P value of <0.05 was considered statistically significant.

## Results

### Serum non-cholesterol sterols as biomarkers of cholesterol metabolism

**Figure 1:**
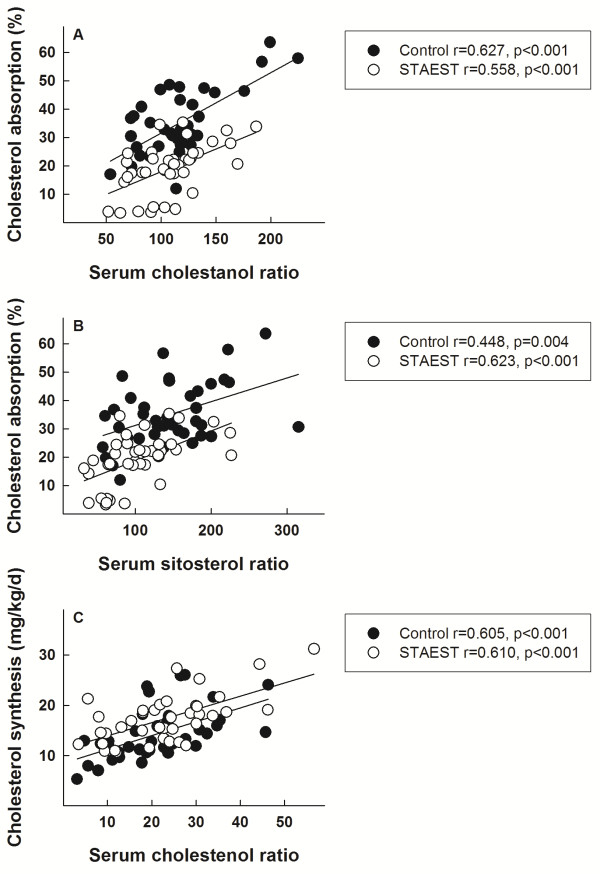
**Cholesterol absorption and synthesis and serum biomarkers.** Three studies [20,27,29] combined, n=39. Panel **A**: Fractional cholesterol absorption (%) and serum cholestanol/cholesterol- ratio (10^2^ × μmol/mmol of cholesterol) in 39 subjects during the control (closed circles) and plant stanol ester (STAEST) (open circles) intervention periods. The results from three studies [20,27,29] were combined. Panel **B**: Fractional cholesterol absorption (%) and serum sitosterol/cholesterol- ratio (10^2^ × μmol/mmol of cholesterol) in 39 subjects during the control (closed circles) and plant stanol ester (STAEST) (open circles) intervention periods. The results from three studies [20,27,29] were combined. Panel **C**: Cholesterol synthesis (mg/kg/d) and serum cholestenol/cholesterol- ratio (10^2^ × μmol/mmol of cholesterol) in 39 subjects during the control (open circles) and plant stanol ester (STAEST) (open circles) intervention periods.

In three studies (n = 39) [20,27,29], cholesterol metabolism was evaluated with simultaneous measurements of fractional cholesterol absorption, cholesterol synthesis, and serum non-cholesterol sterols. During both the control and STAEST periods, serum cholestanol, campesterol and sitosterol concentrations (Table 2) and ratios to cholesterol, shown for cholestanol/cholesterol- and sitosterol/cholesterol-ratios in Figure 1 (Panels A and B), correlated with fractional cholesterol absorption. Similarly, during the control and STAEST periods, serum cholestenol, desmosterol, and lathosterol concentrations (Table 2) and ratios to cholesterol (shown for cholestenol/cholesterol-ratio in Figure 1 Panel C) correlated with cholesterol synthesis. Serum squalene concentration or squalene/cholesterol-ratio did not correlate with cholesterol synthesis. The sitosterol/lathosterol-ratio correlated with fractional cholesterol absorption during the control (r = 0.589, p < 0.001) and STAEST periods (r = 0.662, p < 0.001), and the corresponding values for cholesterol synthesis were r = −0.532, p < 0.001 and r = −0.512, p < 0.001.

**Table 2:** Correlation coefficients between serum non-cholesterol sterol concentrations and the absolute measurements of cholesterol metabolism

**Serum squalene and non-cholesterol sterols, μg/dl**	**During control period**	**During plant stanol ester intervention**
**Cholesterol synthesis, mg/kg/d**		
Squalene	−0.133	−0.053
Cholestenol	0.515**	0.592**
Desmosterol	0.325*	0.425**
Lathosterol	0.366*	0.378*
**Fractional cholesterol absorption, %**		
Cholestanol	0.627***	0.558***
Campesterol	0.394*	0.411**
Sitosterol	0.448**	0.623***

The results from three studies [20,27,29] were combined.

*p < 0.05; **p < 0.01; ***p < 0.001.

N = 39.

### Relative changes in LDL cholesterol and serum non-cholesterol sterol levels

**Figure 2:**
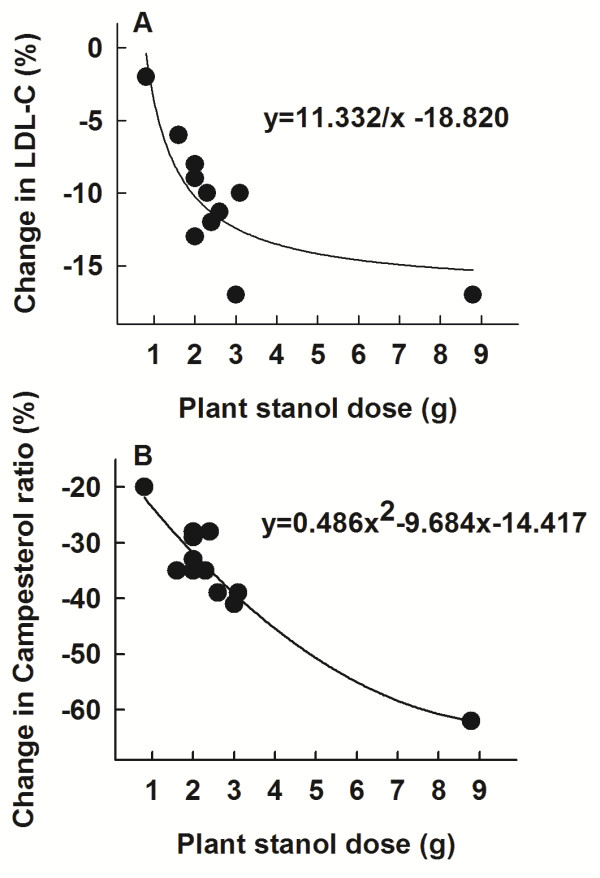
**Changes in LDL cholesterol concentration and serum campesterol/cholesterol-ratio and plant stanol dose.** Panel **A**. Change (%) in LDL cholesterol concentration from controls during the different plant stanol doses in nine studies [17-25]. r = 0.686, p = 0.014. Panel **B**. Change (%) in serum campesterol/cholesterol-ratio from controls during the different plant stanol doses in nine studies [17-25]. r = −0.919, p < 0.001.

In studies involving daily plant stanol intake ≥1.6 g, the LDL cholesterol concentration was significantly reduced (Table 1). Depending on the dose, the LDL cholesterol level was reduced by 6% (plant stanols 1.6 g/d) up to 17% (plant stanols 8.8 g/d). The extent of the reduction in LDL cholesterol correlated with the plant stanol dose (r = 0.686, p = 0.014) (Figure 2 Panel A).

When compared to the controls, serum campesterol/cholesterol-ratio and sitosterol/cholesterol-ratio were reduced in every study (Table 1). The reductions of plant sterol/cholesterol-ratios were dose-dependent as shown for the serum campesterol/cholesterol-ratio in Figure 2 Panel B (r = −0.919, p < 0.001). On the contrary, serum cholestanol/cholesterol-ratio was reduced only in 5/17 trials (Table 1), and the reductions were not associated with the plant stanol dose.

Regarding the serum cholesterol precursors, STAEST consumption increased serum squalene/cholesterol-ratio in 1/17 trials, the cholestenol/cholesterol-ratio in 9/17 trials, the desmosterol/cholesterol-ratio in 8/17 trials, and the lathosterol/cholesterol-ratio in a clear majority in 13/17 of the trials (Table 1). The increase in the precursor sterol to cholesterol ratios varied from 7 to 34%, respectively, but there was extensive variation between different studies even with the same plant stanol dose. In two studies [17,28], all of the precursors remained unchanged.

## Discussion

The new results emerging from these analyses demonstrate that during plant stanol consumption, serum non-cholesterol sterol/cholesterol- ratios reflect the fractional absorption and whole-body synthesis of cholesterol. In addition, serum absorption/synthesis marker ratios were found to be valid markers of cholesterol metabolism during plant stanol intervention. Second, serum levels of plant sterols were dose-dependently reduced by plant stanols. The serum cholestanol level was reduced less frequently than the serum plant sterol levels during consumption of STAEST. The cholesterol precursor sterols, cholestenol, desmosterol and lathosterol, were not increased similarly or were not increased at all in some studies emphasizing the importance of adopting more than one surrogate serum marker. The serum squalene level was not related to cholesterol metabolism.

The study population contained all randomized controlled plant stanol interventions in adults gathered from PubMed, in which at least two serum synthesis markers, two serum plant sterols, and serum cholestanol were analysed. It turned out that the requirement for the non-cholesterol sterols excluded other than our own studies. In fact, 4 more studies could be retrieved from PubMed containing serum lathosterol and plant sterol quantifications, but not the other non-cholesterol sterols.

If one adopts surrogate serum markers, there is always the caveat that their validity should not be considered as self-evident [8]. In situations when there is interference with the homeostasis of cholesterol or the metabolism of an individual marker, the serum value of the marker/s no longer reflects cholesterol metabolism. Accordingly, the metabolism of both cholesterol and the individual marker need to be taken into consideration when interpreting the data. A classic example of potential misinterpretation is the fact that plant sterols cannot be used as cholesterol absorption markers at the same time when the subjects are consuming plant sterol-added products. Similarly, when dietary intake of cholesterol was increased by adding daily egg consumption, there was an elevation in the serum lathosterol concentration, but this no longer reflected cholesterol synthesis but instead the increased intake of lathosterol present in the egg yolk [11]. Even under baseline situations, the relative serum markers may not reflect cholesterol metabolism. In vegetarians, cholesterol synthesis is known to be elevated as compared with control subjects, but there was no increase in the concentrations of the serum cholesterol precursors [33].

In the present study, all serum cholesterol precursors were unchanged in two studies. The subjects in one of these studies were type 1 diabetics, and the precursor ratios to cholesterol varied from −30% to +2%. Why in type 1 diabetes cholesterol synthesis seems not to be activated remains open and warrants further investigation. Regarding the second study, the cholesterol precursors were increased from 14% to 30%, but the increment did not reach significance.

Cholestanol is a saturated derivative of cholesterol produced by the liver. Its synthesis involves mainly a rate-limiting oxidation pathway, but a smaller fraction is also synthesized as a by-product of bile acid production [34]. The dietary intake of cholestanol is minimal, less than 2 mg/d [9], but if biliary secretion is impaired such as in cholestasis, then serum cholestanol values rapidly increase and no longer reflect the absorption efficiency of cholesterol [35]. Even though the serum cholestanol concentration and the cholestanol/cholesterol-ratio correlated with fractional cholesterol absorption during STAEST, in the individual studies the cholestanol/cholesterol-ratio was less frequently reduced than the serum plant sterol/cholesterol-ratios.

In the present analysis, the relative LDL cholesterol reduction varied from 6 to 17% depending on the plant stanol dose used in the different studies. Similarly, the relative reduction for campesterol/cholesterol-ratio varied from 35 to 62%. One can calculate that a 2 g plant stanol dose can achieve about a 10% reduction in the LDL cholesterol concentration and a 32% decrease in the serum campesterol/cholesterol-ratio (Figure 2). Accordingly, the change in serum campesterol/cholesterol-ratio can also be utilized as an indicator of the compliance of the STAEST intake. An earlier study demonstrated that when LDL cholesterol was lowered by 10% with plant stanols, fractional cholesterol absorption was reduced by 45% and the serum campesterol/cholesterol-ratio by 34% (p < 0.05 for all) [36], which is the same magnitude estimated in the present study. These values suggest that approximately a four-fold inhibition in cholesterol absorption efficiency is needed to achieve a unit decrease in the LDL cholesterol concentration.

## Conclusions

In conclusion, serum non-cholesterol sterols are valid markers of cholesterol absorption and synthesis during cholesterol absorption inhibition with plant stanol ester. Serum levels of non-cholesterol sterols did not change consistently in the individual studies emphasizing the importance of utilizing more than one surrogate serum marker. Serum plant sterols decrease dose-dependently after consumption of plant stanols suggesting that the higher the plant stanol dose, the more cholesterol absorption will be inhibited and the more efficiently the LDL cholesterol levels will be reduced.

## Abbreviations

GLC: Gas–liquid chromatography; STAEST: Plant stanol ester.

## Competing interests

HG has received research funding from Raisio Group, Finland. MH and PS declare no competing interests.

## Authors’ contributions

MH collected the data, performed the statistical analysis and drafted the first version of the manuscript. MH, PS, and HG critically revised the manuscript and approved the final version.
